# Coherent Off-Axis Terahertz Tomography with a Multi-Channel Array and f-theta Optics

**DOI:** 10.3390/s24020529

**Published:** 2024-01-15

**Authors:** Karl Henrik May, Shiva Mohammadzadeh, Andreas Keil, Georg von Freymann, Fabian Friederich

**Affiliations:** 1 Fraunhofer Insititute for Industrial Mathematics ITWM, 67663 Kaiserslautern, Germany; 2 Department of Physics and Research Center OPTIMAS, University of Kaiserslautern-Landau, 67663 Kaiserslautern, Germany; 3 Becker Photonik GmbH, 32429 Minden, Germany

**Keywords:** terahertz radiation, computed tomography, detector array, FMCW, acquisition speed, non-destructive testing, a priori information, off-axis measurement, f-theta lens, optics

## Abstract

Terahertz tomography is a promising method among non-destructive inspection techniques to detect faults and defects in dielectric samples. Recently, image quality was improved significantly through the incorporation of *a priori* information and off-axis data. However, this improvement has come at the cost of increased measurement time. To aim toward industrial applications, it is therefore necessary to speed up the measurement by parallelizing the data acquisition employing multi-channel setups. In this work, we present two tomographic frequency-modulated continuous wave (FMCW) systems working at a bandwidth of 230–320 GHz, equipped with an eight-channel detector array, and we compare their imaging results with those of a single-pixel setup. While in the first system the additional channels are used exclusively to detect radiation refracted by the sample, the second system features an f-θ lens, focusing the beam at different positions on its flat focal plane, and thus utilizing the whole detector array directly. The usage of the f-θ lens in combination with a scanning mirror eliminates the necessity of the formerly used slow translation of a single-pixel transmitter. This opens up the potential for a significant increase in acquisition speed, in our case by a factor of four to five, respectively.

## 1. Introduction

Terahertz imaging is a versatile technique in the field of non-destructive testing and imaging [1,2,3]. Possible application scenarios include material characterization [4,5], layer thickness determination [6], as well as moisture and liquid detection [7], spanning various fields of industry, such as automotive, aviation, polymers, petrochemicals, pharmaceutical industry, and many more [1,8,9,10,11,12,13,14,15,16]. Most imaging setups employ raster-like scanning from one side, either in transmission or reflection geometry, to create volumetric sample representations. If the sample under investigation features complex structures, optical effects can lead to image distortion, artifacts, and ambiguity of feature size. Additional challenges arise when attempting to image concealed areas obscured by these sample features. When inspecting additively manufactured [17] or extruded plastic objects [18], such as pipes and window profiles, the application of terahertz transmission tomography [19,20,21] can help overcome these limitations of one-sided imaging approaches. The application of terahertz radiation in a tomographic configuration promises visual 3D scans of samples similar to tomographic X-ray scans but without requiring cumbersome radiation protection measures [22]. Additionally, unlike classic X-ray-based imaging methods, phase-sensitive coherent terahertz tomography detects the time of flight of the radiation, allowing a reconstruction of the complex refractive index. This can be beneficial, especially when investigating low-absorbing materials, which do not lower the intensity of a passing signal significantly [23].

**Figure 1:**
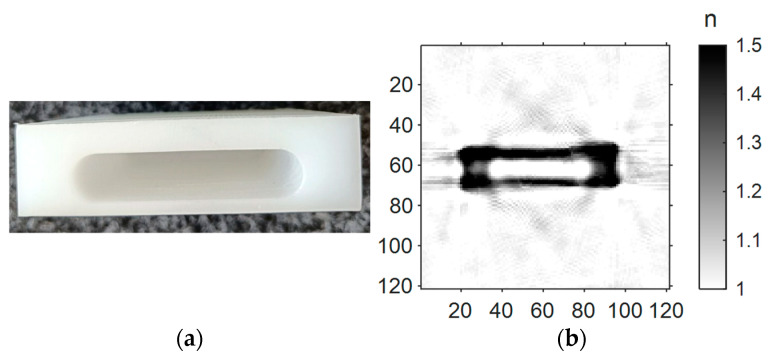
*A priori* information about the sample (**a**), such as outer boundaries and off-axis measurements of the time of flight including deflected radiation, facilitates the tomographic reconstruction of the refractive index (**b**).

In a classical tomographic system using X-ray radiation, projections of an object are acquired by transmitting narrow collimated X-ray beams from various angles through the sample. The radiation travels on usually straight beam paths, allowing for convenient and fast reconstruction using the filtered back-projection algorithm (FBP). Terahertz radiation, on the other hand, has a significantly longer wavelength than X-ray radiation. Consequently, beam collimation leads to comparatively large beam diameters. To detect small features in the sample, one has to use quasi-optical approaches to focus the beam in the center of the imaging scene. Furthermore, the significantly lower photon energy of terahertz radiation in comparison to X-ray leads to a strong interaction between the radiation and the sample material. This interaction includes reflection, refraction, and scattering as well as absorption and a change in the time of flight of the radiation. While the latter two are essential for the tomographic imaging principle, the former lead to a deviation of the terahertz beams from their originally straight paths. These optical effects, if not accounted for in the reconstruction scheme, result in image distortion and artifacts. To anticipate this, we designed a tomographic single-pixel setup featuring one transmitter and one independently moveable detector, allowing the detection of off-axis radiation deflected by the sample [24]. To be able to include the data from the off-axis measurements and additional *a priori* information about the sample in the reconstruction process, we implemented a flexible, iterative, and fast algorithm, the conjugate gradient least squares reconstruction algorithm [23]. The imaging results can be observed in Figure 1 and [24].

The good reconstruction results from the off-axis data come at the cost of very long acquisition times since the entire detection plane must be raster-scanned for deflected radiation for each transmitter position. The acquisition time for the B-scan presented in Figure 1 exceeds 8 h. Since acquisition times of this magnitude are unsuitable for potential industrial applications, we propose the use of a multi-channel array to acquire multiple data points simultaneously and thus speed up the scanning process. In this work, we demonstrate the first terahertz tomography setup featuring an eight-channel detector array. To ensure that enough power enters the small detector aperture, creating high-contrast images with a good resolution, it is necessary to ensure a high-quality focused beam from the transmitter. Previous work has shown the influence of the beam shape on terahertz tomography, and the enlargement of the depth-of-field using Bessel beams [25,26].

In our case, we equipped the system with a telecentric f-θ lens, serving two purposes: firstly, this creates a Gaussian beam focused in the center of the imaging scene, which delivers a trade-off between high image resolution and depth of field. Secondly, in combination with a rotary scanning mirror, the quasi-optical lens allows for a fast scan of the detector plane, addressing the full detector array by simply rotating the mirror. All in all, this yields a significant increase in the scanning velocity.

## 2. Materials and Methods

**Figure 2:**
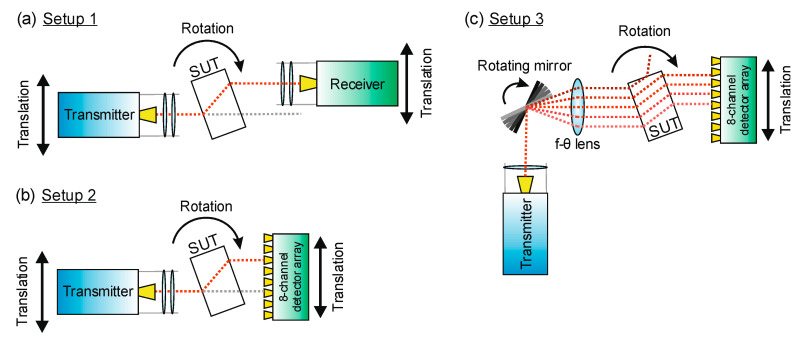
Tomographic setups compared in this work: (**a**) The single-pixel setup with a single transmitter and receiver unit which are moveable independently from each other. (**b**) In Setup 2, the single receiver pixel is replaced by an 8-channel array, increasing the number of pixels acquired at a time. In (**c**), the necessity of moving the transmitter is circumvented with a combination of a scanning mirror and an f-θ lens replacing the focusing lens of the transmitter optics. The (red) dotted lines represent the trajectories of (refracted) beams of radiation.

The three setups covered in this work are depicted in Figure 2. The single-pixel setup (Setup 1, Figure 2a) features one transmitter and one receiver applying the frequency-modulated continuous wave (FMCW) principle to emit and detect terahertz radiation sweeping in a range from 230 to 320 GHz. They are equipped with Pickett-Potter horn antennas emitting a beam, which is collimated by a PTFE lens (f=50 mm) and focused to the center of rotation by a second f=200 mm PTFE lens. The lens configuration shapes the beam approximately into a Gaussian beam with a beam waist of $w_{0}=5.2\;mm$. The receiver unit, which features the same lens setup, mixes the detected radiation with a local oscillator signal, coherently acquiring the signal amplitude and phase. This allows a determination of a sample’s full refractive index from the measured amplitude reduction and time delay of the signal. The single-pixel setup is described in more detail in [23,24]. In [24], we presented an algorithm to include *a priori* information and off-axis measurements in the tomographic reconstruction process. The latter requires time-costly scans of the whole detector plane, which have to be acquired by moving the single-pixel detector along the whole range of motion for every transmitter position and angle. To reduce this acquisition time, we developed Setups 2 and 3.

**Figure 3:**
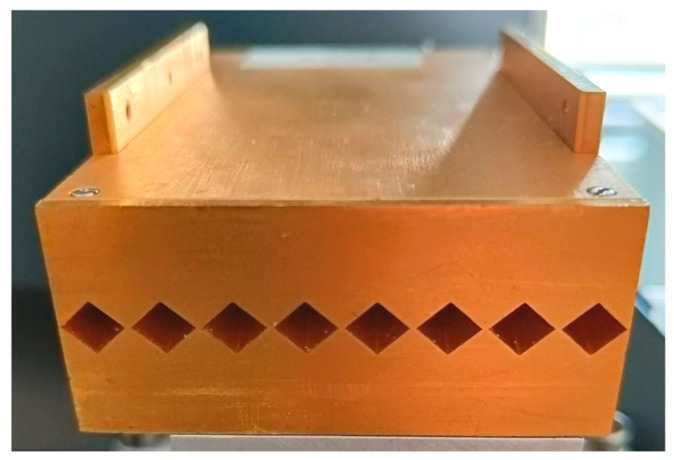
The detector array employed in Setup 2 and 3 features eight independent channels with one antenna and one homodyne mixer each. It is fed with a local oscillator signal and performs coherent detection of eight pixels simultaneously. The width of each antenna is 8 mm, so that the whole array spans 64 mm in total.

In Setup 2, displayed in Figure 2b, the receiver unit is replaced with an eight-channel detector array, operating in the same frequency range as Setup 1 (230 to 320 GHz) [27]. A photograph of the eight-channel array is presented in Figure 3. Analogous to the single-pixel receiver in Setup 1, each channel features a Pickett-Potter horn antenna, which channels the received terahertz signal to a subharmonic mixer fed secondly with a local oscillator signal stemming from the voltage-controlled oscillator in the transmitter. One local oscillator signal is sufficient to allow the simultaneous homodyne signal acquisition of eight detection channels. With this setup, we can potentially achieve an increase in measurement velocity by a factor of eight when acquiring scans of the whole detector plane. However, it still requires a stepped movement of the transmitter. To render this movement of the transmitter unnecessary and increase the imaging speed even further, we introduce a telecentric f-θ lens combined with a rotating scanning mirror, completing Setup 3.

Setup 3, shown in Figure 2c, is designed to keep the transmitter at a fixed position, while still being able to scan the detector plane and address the eight channels of the receiver array quickly. Therefore, after leaving the transmitter, the terahertz beam is collimated and guided by the scanning mirror onto the f-θ lens. The lens design, which is covered in detail in Section 2.1, is optimized to focus the beams in the rotational center of the setup. Thereby, the incident angle θ, on which the beam enters the lens, determines the distance of the focus point from the optical axis. This way, a translation of the focused beam along the detector plane is achieved simply by turning the mirror, allowing the fast acquisition of a projection of the sample.

An important difference between Setup 1 and the following two setups is the lack of focusing optics in front of the detector array, which is difficult to implement due to limited physical space between the densely positioned array antennas. As a result, we expect less directivity of the detector pixels and ultimately a lowered resolution in the reconstructed images as a price to pay for the increased measurement speed.

### 2.1. Design of the f-θ Optics

f-θ lenses are commonly used in many applications involving the displacement of a laser beam for scanning, engraving [28], optical coherence tomography (OCT) [29], and material processing applications. Unlike the common spherical lens, which has a curved focal plane, an f-θ lens provides a flat focal plane with low field curvature, a large field of view, and homogenous beam characteristics in the image plane throughout the entire scan field.

These objective lenses are increasingly applied in microwave and terahertz applications for non-destructive testing, 3D imaging [30], food safety inspection [31,32], etc. Thanks to the non-dispersive characteristics of the available lens fabrication material in terahertz and sub-terahertz, f-θ lenses are incorporated in various transmitters and receivers operating in this frequency region, such as FMCW radars with varying bandwidths [30,33] and TDS systems up to 1.25 THz [34,35].

**Figure 4:**
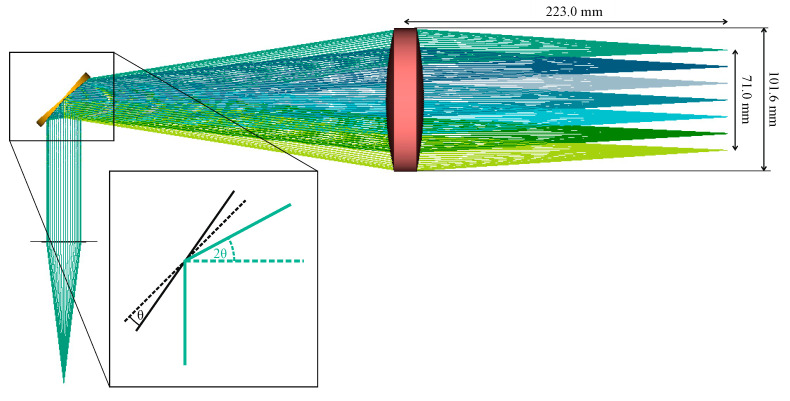
Three-dimensional representation of the Zemax ray-tracing simulation of the telecentric f-θ lens and the rotating scanning mirror. The inlet shows the angle definitions at the mirror.

The f-θ lens employed in this work is designed and optimized for an effective focal length of f=223 mm, a diameter of d=102 mm, and a scan line of $L_{max}=71\;mm$, as shown in Figure 4. It is fabricated out of high-density polyethylene (HDPE) using computer numerical control (CNC) milling. The refractive index and the absorption coefficient of HDPE do not vary significantly in the frequency range in use (230 to 320 GHz), as shown in [33]. In their work, Mohammadzadeh et al. applied a similar lens made from HDPE in an optical setup with a bandwidth of 1.65 GHz. Due to this absence of dispersion in the terahertz spectrum, the lens’s focal length and focus position do not change when sweeping through this frequency range.

The scanning mirror moves θ=±4.5°, deflecting the beam onto the f-θ lens with 2θ=±9°. The displacement of the probing beam from the optical axis is proportional to the incident angle θ:

(1) L=2θ⋅f

Two objectives were the main criteria for the design of the lens:

1. Creating a flat focal plane with telecentricity in the image plane, which guarantees a normal incidence of the focused beam onto the sample;
2. Ensuring that the focus at each measurement point is as small as possible, ideally only limited by diffraction, and for the beam shape to be consistent throughout the mirror angles θ.

The lens design includes aspherical surfaces to eliminate geometrical aberrations. Before entering the f-θ lens, the beam is collimated using an f=10 cm lens. The beam size after collimation was measured to be approximately 32 mm. We assessed the quasi-optical performance of the objective lens by validating the Gaussian beam propagation. To do so, we simulated the optical behavior of the f-θ lens with the optical design software Zemax 13, modeling the horn antenna and the collimating lens as ideal components.

**Figure 5:**
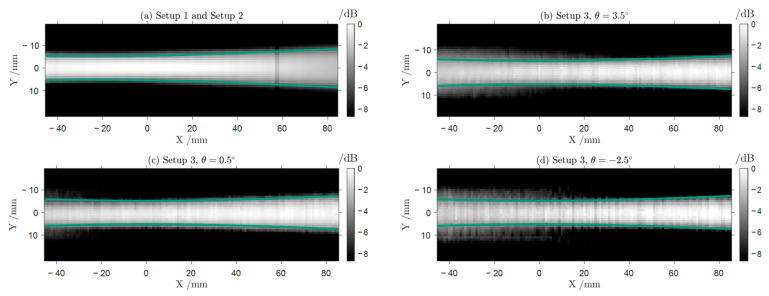
X-Y intensity scans of the beams created by the different quasi-optical configurations. The green lines indicate the boundaries of a Gaussian beam (defined by an intensity decay to $1/e^{2}$ of the intensity on the beam axis) for a wavelength of λ = 1 mm fitted to the data. (**a**) shows the beam created with the biconvex lens employed in Setups 1 and 2. The beam width at the focus of the fitted Gaussian beam is $w_{0}^{1,2}=5.2\;mm$. (**b**–**d**) show the beams shaped by the f-θ lens at different mirror positions. In general, the Gaussian beam is achieved, especially for positive X-values where the detector is positioned. The results of the fitted Gaussian beams indicate a consistent but slightly wider beam width of $w_{0}^{3}=5.5\;mm$.

In Figure 5, we compare raster scans of Gaussian beams created by the f-θ lens under incidence from different angles θ with a scan of the reference beam produced by the standard PTFE focusing lens employed in Setups 1 and 2. The raster scan images in Figure 5 are acquired by moving the detector array stepwise along the beam axis and perpendicular to it. As expected, the beam focus lies in the origin of the diagram, the abscissa representing the direction of the beam propagation. The beam waist is defined by a decay of intensity to $1/e^{2}$ (indicated by the green lines in the figure) and is consistent throughout the range of θ. With values of around $w_{0}^{3}=5.5\;mm$, the measured focus beam widths of the Gaussian beams leaving the f-θ lens are slightly wider than the beam width of the reference beam of $w_{0}^{1,2}=5.2\;mm$ (Figure 5a). As an example, we show three images of the beams for mirror angles between $\theta_{\left(a\right)}=3.5{}^{\circ}$ and $\theta_{\left(c\right)}=-2.5{}^{\circ}$ in Figure 5b–d. The difference in beam width between the systems presumably resulted in a lowered resolution in the reconstructed images. Especially for very eccentric mirror positions, the beams tend to widen slightly before the focus (X≤0 mm) in Figure 5b,d. Nevertheless, for X≥0 mm, all beams show an intensity distribution that is consistent with the Gaussian beam approximation with $w_{0}^{3}=5.5\;\mathrm{mm}$. This indicates that the f-θ lens enables a scan of the imaging scene with beams focused in the imaging plane, which can be successfully described by the Gaussian beam model.

### 2.2. Image Reconstruction Process

The image reconstruction employed in this work has been discussed at length in [23], and the incorporation procedure of *a priori* information is covered comprehensively in [24]. Here, we will therefore only give a brief introduction to the aforementioned concepts. The reconstruction algorithm is based on the conjugate gradient least squares algorithm (CGLS) [36]. It is a fast, versatile technique to approximate iteratively the solution to the inverse problem

(2) $$A\cdot \vec{x}=\vec{b}$$

In this case, $\vec{x}$ represents the unknown image vector and $\vec{b}$ stands for the measured data acquired in the measurement process, also referred to as a sinogram. The matrix A models the path on which the probing beam of terahertz radiation travels through the imaging scene. When on this path $\vec{r}$, the beam interacts with an object, which has a refractive index of

(3) $$\tilde{n}=n+in\prime \prime ,$$

differing from the refractive index of air ${\tilde{n}}_{air}\approx 1+0i$. The time of flight (TOF) is directly proportional to the real refractive index

(4) $$T=\frac{1}{c_{0}}\int \left(n-n_{air}\right)d\vec{r},$$

whereas the relative intensity loss $\tau =\left(\frac{I(\vec{r})}{I_{0}}\right)$ is according to Lambert-Beer’s law:

(5) $$\tau =\exp\left(-\int \frac{4{\pi n}^{\prime \prime }f}{c_{0}}d\vec{r}\right).$$

Inserting the definition of the absorption coefficient $\alpha =4{\pi n}^{\prime \prime }f/c_{0}$, we can express the intensity loss in its logarithmic representation as:

(6) $$\tau_{ln}=\ln\left(\tau \right)=-\int \alpha d\vec{r}.$$

Discretizing the imaging scene $\vec{I}$ as a square area of $p^{2}$ pixels, (4) and (6) become

(7) $$T_{B}=\frac{1}{c_{0}}\sum_{i\in B}\left(n^{\prime }\left(x_{i}\right)-n_{air}\right)x_{i}=\sum_{i\in I}{\vec{A}}_{i}\cdot {\vec{I}}^{T},$$

(8) $$\tau_{B}=-\sum_{i\in B}\alpha \left(x_{i}\right)x_{i}=\sum_{i\in I}{\vec{A}}_{i}\cdot {\vec{I}}^{\tau }.$$

Here, $x_{i}$ represent the length of the path on which the beam B crosses pixel i. n and α are assumed to be constant within one pixel. The column vectors ${\vec{A}}_{i}$ of the matrix A are populated with the $x_{i}$ values, determining which pixels contribute to which beam to which extent. This way, by inserting the measured TOF values $T_{B}$ into the sinogram vector $\vec{b}$ in (2), we can solve ${\vec{b}}_{T}=A\cdot {\vec{I}}^{T}$ for the real refractive index distribution ${\vec{I}}^{T}$ of the imaging scene, and ${\vec{b}}_{\tau }=A\cdot {\vec{I}}^{\tau }$ for the image showing the absorption coefficient, respectively. 

**Figure 6:**
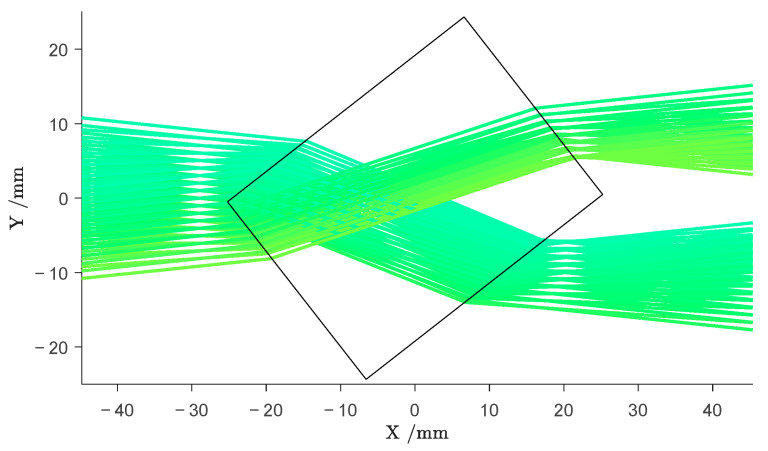
Ray-tracing simulation of a probing beam passing through a rectangular object. Including *a priori* information about the sample in the reconstruction process significantly improves the image quality.

The flexibility of the CGLS algorithm comes with the possibility to freely design the Matrix A. As thoroughly explained in [24], this opens up the opportunity to incorporate *a priori* information into the tomographic reconstruction process, enabling a more precise modeling of the measurement process. While the above model of the interaction between the beam and the sample is based on the assumption that the probing beams had a vanishing diameter, it is in fact a Gaussian beam with a diameter relevant to the feature size of the objects (see Section 2.1). Ignoring this leads to significant distortions and reconstruction errors in the image [24]. We were able to incorporate the non-vanishing beam size into the matrix A by considering one beam as a combination of one-dimensional infinitely thin rays with different starting positions and directions (see Figure 6). This model describes the probing beam more accurately but preserves the linear relation between the sinogram values and the image pixels in (7) and (8).

The second set of *a priori* information we consider in the reconstruction process is the shape of the sample and its refractive index. In the field of NDT, this is a relevant scenario, since the desired shape and material of a sample are often known *a priori*. Since the wavelength of the terahertz radiation in use is in the same order of magnitude as the features to be observed, optical effects occurring at the interfaces between the sample and the surrounding air can influence the beam propagation severely. These optical effects include refraction and reflection, which alter the beam propagation directions according to Snell’s law. Considering the outer boundaries of a sample as well as its refractive index, one can apply ray tracing to predict the ray paths on which the radiation travels through the imaging scene (see Figure 6).

Figure 6 illustrates that for certain sample geometries and angles, the probing radiation can deviate significantly from its originally straight path. Nevertheless, to account for the deviated radiation in the reconstruction process, we conduct off-axis measurements; i.e., scanning the entire detector plane for deviated radiation. From the ray-tracing simulations, we determine the position of the highest intensity and include the values measured at this position in the reconstruction as described in [24]. While this procedure increases the quality of the reconstructed images, it renders the acquisition very time-consuming when working with Setup 1, because one has to scan the whole detector plane stepwise or in a continuous movement for every transmitter position (and rotation angle).

Hence, we developed Setup 2 and finally Setup 3 to reduce the acquisition time. The former facilitates the simultaneous acquisition of data at eight different positions in the detection plane. The latter allows a continuous acquisition at eight different positions in the detector plane, while a rotation of the mirror provides scanning beams moving through the emitter plane.

## 3. Results

**Figure 7:**
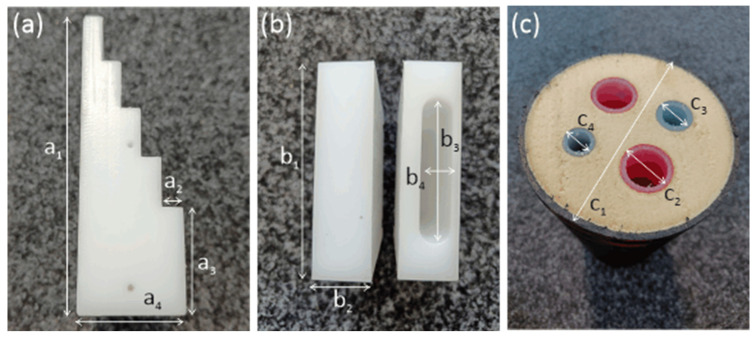
Samples 1–4 imaged in this work: (**a**) Sample 1: Step wedge made from polyethylene (PE), (**b**) Samples 2 and 3: PE cuboids; Sample 3 has a stadium-shaped drilled-out hole (**c**) Sample 4: district heating pipe with four inner pipes. The indicated dimensions can be found in Table 1.

To test and compare the imaging capabilities of the three setups presented in Figure 2, we designed four samples, shown in Figure 7. The first three samples are made of polyethylene (PE), which has a real refractive index of $n_{PE}=1.52$ and a very low absorption coefficient of $\alpha_{PE}<0.05\;{mm}^{-1}$ [5]. While Sample 1 is a stepped wedge, Samples 2 and 3 have a rectangular base shape. In Sample 3, we milled an obround (i.e., pill-shaped) hole. Sample 4 is a piece of a district heating pipe, consisting of four inner tubes made from plastic and a filling of porous foam. An outer layer of plastic protects the foam filling of the pipe. The outer layer has an undulating structure, so that its diameter ($c_{1}$ in Figure 7c) varies in a range between 137 mm and 143 mm. The specific materials used for production are protected intellectual property. We were able to determine the real refractive index of the tubes and outer layer as $n_{4}\approx 1.6$. The absorption coefficient is approximately $\alpha_{4}\approx 0.5\;mm^{-1}$. The foam filling has an almost vanishing absorption coefficient and a real refractive index close to 1. Since it does not interact stronger with radiation in the terahertz range than air, its influence can be neglected. The dimensions of the samples are given in Table 1. All samples were scanned by acquiring between 120 mm and 200 mm wide projections with a pixel size of 1 mm from different directions, turning the sample with an angle step size of 2°.

**Figure 8:**
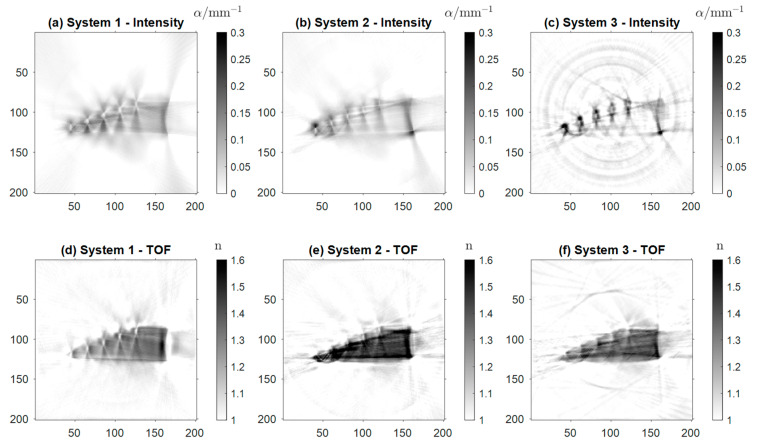
Reconstructions of Sample 1, (**a**–**c**) representing the absorption coefficient α and (**d**–**f**) the real refractive index *n* created with Setups 1, 2, and 3.

Figure 8 displays the images of the first sample, created with the three systems. Figure 8a–c shows the reconstructions of the absorption coefficient α from the intensity sinograms. Given the low absorption coefficient of PE, absorption within the sample is minimal. The changes in the measured intensity are dominated by optical effects occurring at the sample edges [24]. Consequently, despite *a priori* consideration of these effects, the edges of the sample still appear much too prominently in the reconstruction. This hinders a quantitative analysis of the object’s absorption coefficient consistently throughout all three measurement setups. For Samples 2 and 3 we therefore refrain from presenting the reconstructions resulting from the intensity data. On the other hand, the images displayed in Figure 8d–f clearly depict the shape of Sample 1 and accurately determine its real refractive index n. Between the images created with the different systems, there were only subtle differences in reconstruction quality or resolution. The reconstruction acquired with Setup 2 (Figure 8e) shows a stronger contrast than the other two. Especially in comparison with Figure 8f (Setup 3), it exhibits a slightly better resolution, due to the smaller focus of the probing beam.

**Figure 9:**
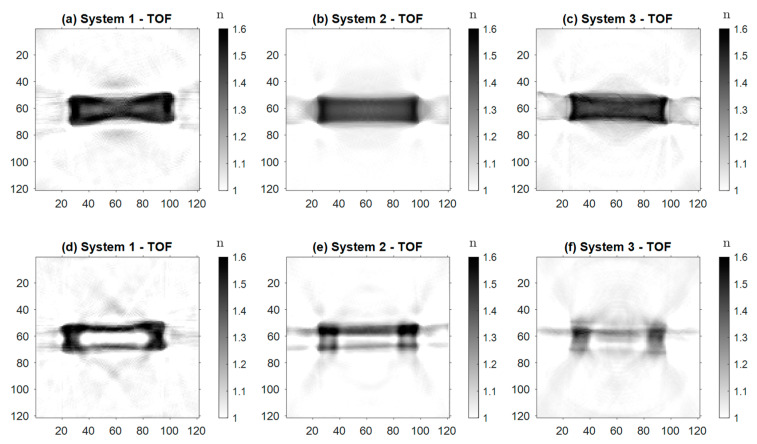
Reconstructions of the real refractive index *n* (**a**–**c**) of Sample 2 and (**d**–**f**) of Sample 3, created with Setups 1, 2, and 3.

The images of Samples 2 and 3 can be observed in Figure 9. All six images display the real refractive index n, reconstructed from the TOF of the radiation passing through the samples. Figure 9a–c correspond to Sample 2. All three systems reconstructed the shape of the sample correctly. The reconstructions created with Setups 2 and 3 (Figure 9b,c) appear sharper and the inner part is more uniform than in Figure 9a. Quantitatively, all three systems correctly reconstructed the refractive index. For Sample 3 (Figure 9d–f), Setup 1 produces the most accurate reconstruction. The image (Figure 9d) is sharper and the shape of the sample—especially the drilled-out hole—is more visible than in the other reconstructions. Nevertheless, in all three images, the shape of the sample and especially the difference in comparison to Sample 2 is distinguishable, along with the quantitative value of the real refractive index n of Sample 3.

**Figure 10:**
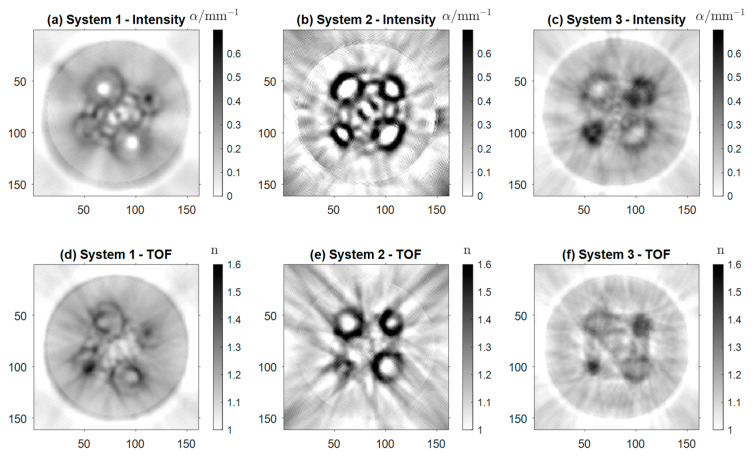
Reconstructions of Sample 4, (**a**–**c**) representing the absorption coefficient α and (**d**–**f**) the real refractive index *n* created with Setups 1, 2, and 3.

Figure 10 shows the reconstructions of the district heating pipe (Sample 4). Figure 10a–c display the absorption coefficient α of Sample 4 based on intensity projections, while Figure 10d–f show the real refractive index n. In Figure 10a, the shape of the pipe as well as the foam filling are clearly noticeable. Presumably, this is more likely a result of the scattering properties of the foam than its absorption. The four inner tubes are visible. In the case of the two larger tubes, one can also distinguish the air core from the wall. However, for the smaller tubes, the inner wall is reconstructed as a single dark spot, giving the appearance of high absorption. Again, this is the result of scattering and strong refraction of the beams in the small tube core. The artifacts resembling concentric circles of low and high intensity are the cause of multiple reflections between the tubes.

The same effect can be observed between the inner tubes in Figure 10b. However, here all four air cores of the inner tubes are distinguishable from the tube walls. The background noise in the image is significantly higher than in the images from the other two systems, due to the lack of focusing optics of the detector. Additionally, the contrast and the background noise of the image seem to be higher than in Figure 10a. In the reconstruction acquired with Setup 3 (Figure 10c), all four tubes are detectable, the air cores of the two biggest tubes being distinguishable. The artifacts between the tubes are reduced, and the outer tube and the foam are visible. Overall, the resolution is lower in Figure 10b,c due to the aforementioned lack of focusing optics for the detector array. The same holds for the reconstructions of the real refractive index n. Although Figure 10d–f are less detailed than their counterparts based on the intensity projections, all four tubes are identifiable in the images. The smaller tube radii of the small tubes lead to stronger refraction of the radiation. As a result, the small tubes are not fully resolved and appear to have a larger absorption coefficient than the two larger tubes. In Figure 10d, the reconstruction acquired with Setup 1, the inner and outer walls of the tubes are visible, as well as the general shape of the pipe. In Figure 10e,f, strong artifacts of the inner tubes govern the reconstructions. Nevertheless, in Figure 10e, the air cores are again visible for all four tubes, and their shape is reconstructed more accurately than in Figure 10b. In Figure 10f, there are strong artifacts between the tubes, whose cores are barely visible.

The benefits of Setups 2 and 3 become evident when considering the acquisition times of the sinograms the shown images are reconstructed from. By using the detector array, we reduced the measurement time by a factor of four. Combining it with the scanning mirror and the f-θ optics, we achieved an even faster measurement, up to five times compared to Setup 1.

## 4. Discussion and Outlook

Overall, Setup 1 delivers the best reconstruction images. This can be observed mainly in Figure 9d–f and Figure 10 (Samples 3 and 4). Its main advantage is the focus optics in front of the detector, which yield a decrease in the effective detection aperture below the antenna’s physical aperture and a displacement into the center of the setup. This results in a higher resolution and lower background noise, in contrast to the detector array applied in Setup 2 and Setup 3. Limited physical space resulting from the high receiver density, the small aperture of the detecting antennas, and the comparably large wavelength render the implementation of focusing optics very challenging. The design of quasi-optical elements reducing the effective aperture of the detector array is currently subject to further investigation and could pave the way to eliminating the shortcomings of the newly designed setups.

Another reason for the lower image quality of Samples 3 and 4 is that the amount of *a priori* information incorporated in the reconstruction is lower than for Samples 1 and 2. The inner structure of the samples is not part of the ray tracing simulations. This excludes the drilled-out defects or inner pipes in Samples 3 and 4. The resulting deviations of the beam paths from the simulated ones lead to the blurring of the reconstructed image when acquired with the low aperture detector array.

Comparing Setups 2 and 3, we generally observe a decrease in contrast and resolution when employing the f-θ lens, again, especially for Samples 3 and 4. The decrease in contrast and resolution when employing the f-θ lens can be attributed to its focusing capability, which is the only difference between the two systems. Figure 5 displays the increased focus beam width, and that for large angles θ, i.e., configurations for which the probe beam is displaced fairly far from the optical axis, the beam quality decreases before the focus. As this part of the beam is where the first interaction with the sample occurs, optical effects like refraction can strongly alter the beam paths from the trajectories predicted in the ray tracing simulations. This, in turn, lowers the resolution of the reconstructed image.

Currently, we are aiming towards building a setup, which applies a SiGe-MMIC multi-channel array not only in the detector plane but also as a transmitter unit. We already performed promising preliminary measurements with a system featuring two multi-channel arrays [37]. The system could potentially allow the elimination of any mechanical movement (other than rotating the sample), but the high density and the resulting small aperture of the transmitting antennas lead to strongly diverging emitter characteristics and suboptimal resolution. One potential solution could be digital beamforming [38] to re-establish the directivity of the beams. This could even pave the way to shaping the beam to a Bessel or Airy shape if desired [25,38,39].

## 5. Conclusions

**Figure 11:**
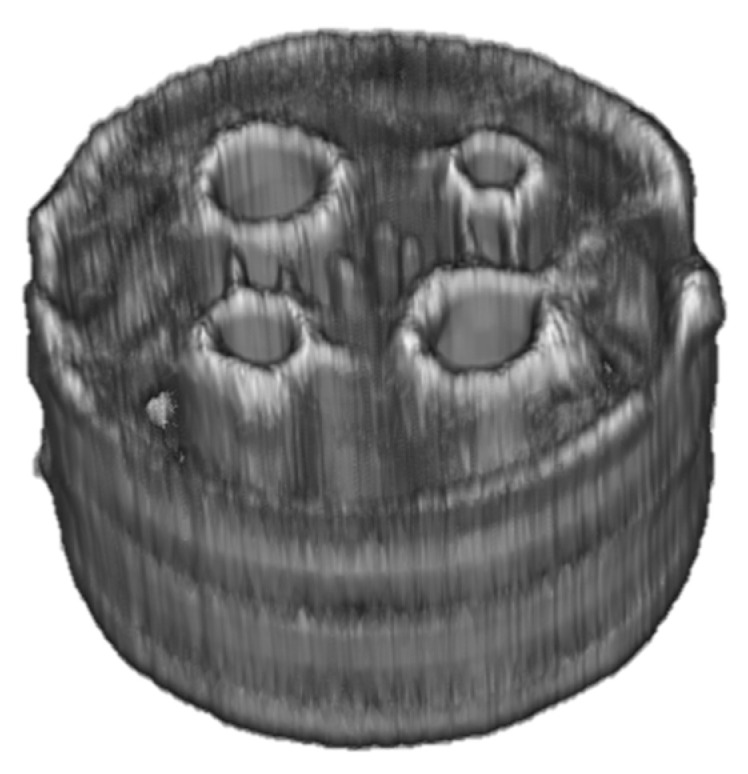
3D image created by vertically stacking multiple cross-sectional scans of Sample 4 acquired with Setup 1.

In this work, we present two novel terahertz tomography setups featuring an eight-channel detector array. To the best of the authors’ knowledge, these are the first tomography setups that allow the simultaneous acquisition of eight sinogram or off-axis measurement pixels in the terahertz range. Therefore, it marks a significant step towards the applicability of terahertz tomography in an industrial context. The application of the eight-channel array offers the opportunity of increasing the measurement speed up to a factor of eight. However, for the setups presented in this work, the necessity of moving the transmitter or the mirror limits this potential increase to a factor of four or five when combined with an f-θ lens. Especially if one seeks to acquire 3D representations of the sample, such as the one of Sample 4 in Figure 11, an increase in measurement speed is crucial. In the current configuration of the systems, this increase comes at the cost of a minor loss in resolution and detail in the reconstructed image.

Overall, each evolution of the systems presented in this work resulted in a slight decrease in imaging quality, serving as a trade-off for an increase in measurement speed. On the other hand, the designs of Setup 2 and 3 mark important steps towards the conceptualization of a stationary setup, based on two chip-based multi-channel arrays, which render mechanical movements of transmitter and receiver unnecessary. With this work, we prepare the foundation for much simpler, faster, and more robust system designs. With the systems presented here and the vision of relying more and more on fully integrated electronic arrays, we are confident we can make terahertz tomography ready for the industrial market in the near future.

## Figures and Tables

**Table 1:** Sample dimensions as indicated in Figure 7.

Sample	Dimension 1	Dimension 2	Dimension 3	Dimension 4
1	$a_{1}=120\;mm$	$a_{2}=8\;mm$	$a_{3}=40\;mm$	$a_{4}=40\;mm$
2 & 3	$b_{1}=75\;mm$	$b_{2}=20\;mm$	$b_{3}=50\;mm$	$b_{4}=10\;mm$
4	$c_{1}=137–143\;mm$	$c_{2}=35\;mm$	$c_{3}=25\;mm$	$c_{4}=20\;mm$

## Data Availability

The data presented in this study are contained within the article.
